# IoT-driven smart irrigation system to improve water use efficiency

**DOI:** 10.1038/s41598-025-33826-6

**Published:** 2026-01-16

**Authors:** Zeinab E. Mohamed, M. K. Afify, M. M. Badr, O. A. Omar

**Affiliations:** 1 Department of Agricultural Engineering, Zagazig University, Zagazig, Sharkia Egypt; 2 Department of Agricultural Engineering, Agricultural Research Center, Zagazig, Sharkia Egypt

**Keywords:** Smart irrigation system, IoT, Sensors, Water use efficiency, Mobile application, Engineering, Environmental sciences, Plant sciences

## Abstract

The agriculture sector is the cornerstone of many global economic entities, plays a central role in highly contributing to ensure food security and the gross domestic product. Difficulties caused by traditional irrigation methods, population growth, and climate change are leading to the development of current irrigation systems. This study presented a smart irrigation system using novel techniques like, Internet of Things (IoT), cloud computing, embedded system and sensors. The smart system integrates real-time monitoring and control during irrigation, fertilization, and biopesticides application. A mobile application is implemented to monitor and control the entire system. Results showed that using wood vinegar at low concentrations is an effective way to improve water use efficiency, increase lettuce yield, and optimize disease control compared to other concentrations. The impact of 400 concentration on the evaluation criteria was found to achieve the best values at 26% moisture content. The smart system reduces water consumption by 47% and achieving a 43% increase in yield as well the lowest level of disease severity index with a value of 7.78%. The system proposed features real-time monitoring and control, improving water use efficiency and supporting smart agriculture practices as well as contribute to food and water security.

## Introduction

Lettuce (Lactuca sativa L.) is a leafy herbaceous annual plant in the family Asteraceae grown for its leaves. The Lettuce plant can vary greatly in shape, size and leaf type generally, the leaves of the plant form a loose rosette or dense head which are used as a salad green. Lettuce is the world’s most popular leafy salad vegetable, primarily for human consumption of their fresh, succulent leaves. The Lettuce plant can grow to a height of 30–90 cm in height and is typically grown as an annual, harvested after only one growing season. The Lettuce plant is rich in various vitamins and minerals, it has benefits such as anti-inflammatory properties, antioxidant properties, antioxidant, low cholesterol level, reduce insomnia, anti-cancer properties and antimicrobial. It is rich in vitamin B-complex, C and K, calcium, potassium, thiamine, folate and riboflavin^1^.

With the proliferation of synthetic intelligence (AI) and the Internet of Things (IoT) technologies, many structures are changing closer to being smart, that is due to the fact the truth that the IoT enabled many structures to be related to the Internet, in which application-unique information may be effortlessly sensed, and the captured information are transmitted over a wi-fi channel to a faraway vacation spot for in addition processing and analysis. Smart agriculture is one instance of those conventional structures which can be changing to clever structures. IoT gadgets may be established in farms in clever agriculture to gather statistics the use of agriculture-associated sensors which include soil moisture, surroundings temperature, humidity, and mild intensity^2^. The approbation of innovative technologies, including the Internet of Things and artificial intelligence, provides new opportunities to enhance precision agriculture practices, with a focus on the role of sensors, cloud platforms, and communication networks in collecting, transmitting, and analyzing data in real-time^3^. Smart irrigation represents a promising solution for water management in precision agriculture. This system improves water use efficiency and yields by integrating real-time soil and climate data, contributing to the resilience of agricultural practices in the face of the growing challenges of water scarcity and the impacts of climate change^4^. Relying on integrated systems and sensors to create a real-time smart irrigation system represents an innovative solution to the growing challenges of water management in agriculture, improving water use efficiency while enhancing agricultural productivity^5^. The integration of blockchain and IoT technologies intends to revolutionize agricultural sector by improving current practices and transforming them into more efficient and sustainable systems^6^. Water management in agriculture is critical, particularly in regions facing water scarcity. Improper irrigation practices can cause overuse of water, growing expenses and lowering crop first-class and yield. Therefore, optimizing the irrigation manner the usage of particular data-pushed predictions is critical for each water conservation and maximizing agricultural output. These parameters play an important function in supporting the farmers domesticate their farms, make medical choices on how tons and while to irrigate the soil, fertilization, pest control and lots of different essential crucial actions and decisions^7^. Internet of Things (IoT) based solutions are proving very helpful in many dimensions of the agricultural landscape, and these intelligent solutions could also be fruitful in smart irrigation with optimum crop management, particularly in fertilization, irrigation and disease diagnoses practices^8^.

Biopesticides is derived from natural materials such as plants, animals, microbes and minerals as a new category of pesticides. Biopesticides are applied to control of parasites and pests in a non-toxic, environmentally friendly manner and sustainable. They are also characterized by long-lasting and safety on non-target organisms, including human health and the environment, where it poses less toxicity compared to chemicals compounds, with little or no residual effects. The application of biopesticides in plant protection leads to many fundamentally beneficial as reducing pesticide residues in food and reduce risks to consumers^9^. Wood vinegar (WV) also known as pyroligneous acid or wood acid; it is a byproduct from charcoal production. It is a liquid generated from the gas and combustion of fresh wood burning in airless condition. When the gas is cooled, it condenses into liquid. The principal components of wood vinegar are acetic acid, acetone and methanol. In addition, the vinegar often contains 80–90% water along with some 200 organic compounds^10^. Wood vinegar improves soil quality, controls plant growth, eliminates pests and toxic to plants if too much is applied. It also accelerates the growth of roots, stems, leaves, improve crop yield, fruit size, and fruit quality^11^. Wood vinegar is rich in bioactive compounds such as acids, esters, sugars, alcohols, and phenols, making it a valuable resource in sustainable agriculture. It has shown promise in enhancing disease resistance and stress tolerance in plants, providing a natural alternative to synthetic chemicals^12,13^. Wood vinegar (WV) enhances crop resistance to diseases, leaf area index, increases crop yield, and reduces disease incidence like downy mildew in plant. WV with its antibacterial, antioxidant and growth-promoting characteristics enhances crop resilience, yield, and quality^14^. It also improves salinity tolerance, indicating that it can improve crop resilience in saline environments by lowering oxidative damage and protecting photosystem^15^.

The existing literature review reveals significant gaps in an intelligent agriculture solution. (1) Current technologies dependent on expensive hardware and software, making them inaccessible for many farmers at the present time. (2) Smart agriculture is often controlled using closed-source software architectures, in other words, there is a noticeable lack of open-source software. (3) There is a lack of use of weather stations in smart farms. (4) Further research is needed to determine optimal concentrations of wood vinegar and its relationship to irrigation water conservation. This research explores an innovative and environmentally friendly technology that enable sustainable crop production by designing a smart IoT-based system for real-time monitoring and control to improve water use efficiency.

The main objective of the study is to evaluate the effectiveness of an IoT-based irrigation monitoring and control system and data collection technology for agricultural applications. Specifically, this study provides the following contributions: (1) Design a smart irrigation system that integrates advanced technologies including IoT technology, cloud computing, embedded systems and effective communication protocols in real-time. (2) ESP32 microcontroller is used to construct low-power sensors and actuators to collect accurate and continuous data. (3) Open-source software is designed to handle data collection, control, and operation. (4) Implement a mobile application based on the SSE protocol to update data automatically and continuously via an easy-to-use interface. (5) Determine the appropriate concentration of wood vinegar to enhance Lettuce growth and save water consumption. These contributions aim to improve the use water efficiency and increase agricultural yield, thereby contributing to promote improved resource sharing, and develop agricultural practices and overall sustainability.

## Methods

The field experiments were carried out for two years (2024 and 2025) at Research Farm, Faculty of Agriculture, Zagazig University, Egypt, having latitude of 28◦38’21.3” N and longitude of 77◦08’56.5” E at an altitude of 14 m above mean sea level. A smart system that uses the Internet of Things (IOT) includes a set of devices that are connected to collect data, analyze that data, as well as effective management of irrigation water in real time. IOT and other device technologies can improve agricultural operations management and make agriculture more environmentally friendly. This system uses advanced technologies such as embedded systems, Internet of Things, HTTP, HTML, CSS and JavaScript. which takes advantage of the programs that are provided by the server. Using the ESP32 microcontroller, the system collects sensor data in real-time and sends it to a web, which can be continuously monitored and informed decision-making.

### Experimental details

The experiment was conducted by growing Lettuce in well-drained pots for two years with three replicates. Information on the weather at the experimental site has been provided, data of temperature, humidity, light intensity and soil moisture were sourced from the sensors as one of basic parts of the proposed system for the experiment, which have been pre-calibrated.

### Smart system

The current study uses specialized sensors to the most vital agricultural related parameters such as the soil moisture content, weather temperature and humidity and light intensity that are directly related to the smart system. The system uses a pump & valves to add water and wood vinegar to the soil and plant according to values determined by irrigation and biopesticides control management algorithm. This system determines the amount of used water that plants need based on real-time IoT sensors. A mobile application was implemented to monitor and control the entire system. This system is water and energy efficient and can be used in both small and large greenhouses.

The architecture of IoT devices is designed to collect environmental data and send it to a central server over a network. For the server to make appropriate decisions based on information from IoT devices that measures temperature, humidity, soil moisture, and water volume in real-time. The key components of this design include the piping network, pump, emitters, and the IoT-connected automated control system. It’s ideal for small and medium-sized agricultural, submerged water laminar flow. The water solenoid valve has two half-inch outlets, normally, the valve is closed and when 5VDC is applied to the two terminals, the valve opens and water can push through, also water can only flow one direction. The Wi-Fi module/Mobile data communication module is used as a connection between the field devices and the server. A Wi-Fi module has been used to transmit data to an HTTP server in the proposed structure.

### Lettuce plant

The Lettuce plant used Romaine or cos Lettuce (*Lactuca sativa L. var. longifolia*) were sourced of the EARI-Egyptian Agricultural Research Institute in Giza, Egypt, with maturity period of 90 days (germination, seedling, vegetative growth and maturation) stage. The Lettuce was manually planted in the middle of October in well-drained cylindrical pots, 30 × 30 cm diameter and depth, three plants were planted per pot.

### Soil preparation

The soil is clay loam texture with 31% clay, 34% silt, 35% sand, and highly compacted below 0.4 m depth, and soil properties are reported in Table 1. Soil moisture ranges are 21, 26, and 32% during the growth stages of Lettuce crop, with rates from 60 to 90% of field capacity. These rates are controlled using soil moisture sensors in the smart system.

**Table 1:** Physical properties of soil, and soil-water relationships.

Sand [%]	Silt [%]	Clay [%]	Texture class	FC [%]	WP [%]	BD [g/cm^3^]
35	34	31	Clay Loam	35.00	15.00	1.45

FC: Field capacity; WP: Wilting point; BD: Bulk Density.

### Wood vinegar

A wood vinegar (WV) branded as an organic alternative in pest control and fertilization operations was used for the experiment. wood vinegar contains acetic acid, organic carbon, hydrocarbons, acetone, propionic acid, phenols, ammonia nitrogen, sulfates, iron, manganese, etc. WV used in the study was chemically characterized as shown in Table 2.

**Table 2:** Chemical and biochemical parameters of wood vinegar.

Parameter	Unit	Value
Acetic acid	mg.l^− 1^	27,840
Total organic carbon	mg.l^− 1^	15,600
Hydrocarbons	mg.l^− 1^	77
Acetone	mg.l^− 1^	320
Propionic acid	mg.l^− 1^	72,447
Phenols	mg.l^− 1^	54
Total suspended solids	mg.l^− 1^	26.0
Ammonia nitrogen	mg.l^− 1^	8.3
Sulfates	mg.l^− 1^	53.7
Sulfites	mg.l^− 1^	0.32
Sulfides	mg.l^− 1^	0.12
Chlorides	mg.l^− 1^	29.2
Total phosphorus	mg.l^− 1^	0.97
Nitrates	mg.l^− 1^	5.2
Aluminum	mg.l^− 1^	0.92
Arsenic	mg.l^− 1^	0.02
Iron	mg.l^− 1^	183
Manganese	mg.l^− 1^	1.11
pH	-	3.2
Electrical conductivity	µS cm^− 1^	1389

Wood vinegar was used to treat plants by injection with drip irrigation after diluting it with water every two weeks. After 15 days of potted Lettuce plants, WV treatments were applied to each treatment at dilution ratios of 1:200, 1:300, 1:400, and 1:500 near the root zone. The treatment process of injection wood vinegar was carried out under both control and monitoring by using the smart system.

### Framework of proposed system

**Fig. 1:**
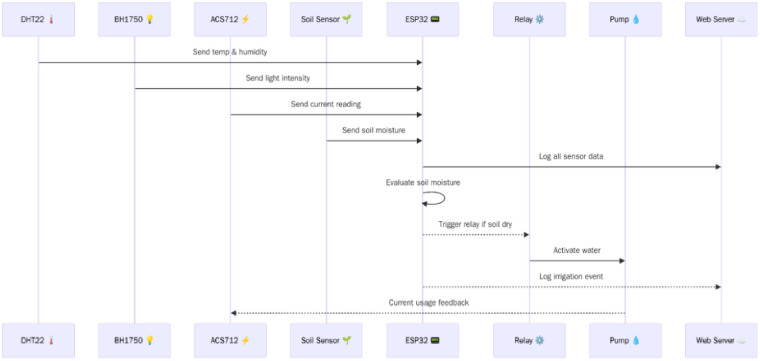
Framework of the proposed smart system.

**Fig. 2:**
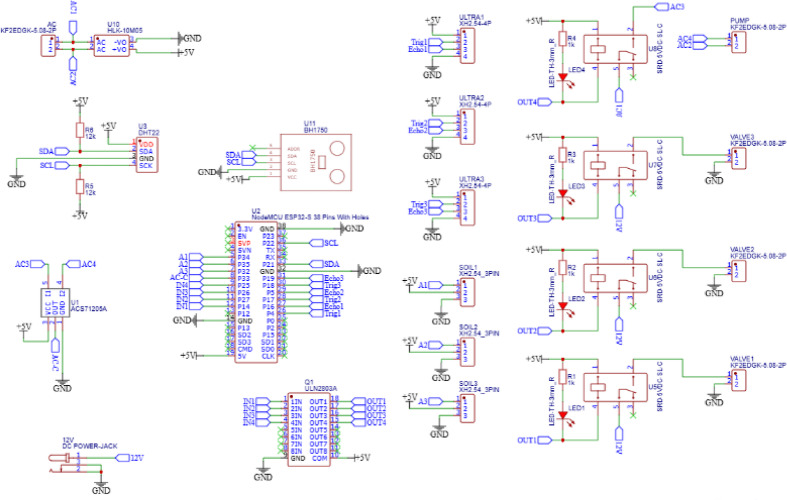
Connection of the board schematic.

This system explicates how ESP32 microcontroller is embedded into a web server for the smart system. Using this microcontroller, the browser can automatically and quickly receive updates from the server over HTTP connection, this is critical for transmitting sensor data in real time. Smart technique is particularly useful in programs that require immediate updates, the general framework for the system can be shown in the following Fig. 1. When a new reading is available, microcontroller sends this data to the client, and the website page is automatically updated without any intervention. The process begins by establishing a connection between the client and server via Server-Sent Events (SSE). This data is then sent to the customer as specific events such as temperature, soil moisture, air humidity, light intensity and water volume, Fig. 2 shows connection of the board schematic.

The application created in this work displays sensor readings as numbers and media counters. The web server is developed using CSS and HTML ensuring an aesthetically attractive and functional user interface. This design allows for real-time monitoring of operating conditions, ensuring efficient and automated management of natural resources. It also provides user-friendly interfaces that enable users to monitor environmental conditions and make informed decisions on agricultural operations. The proposed system in this research optimizes decisions taken based on factors such as crop type, climate conditions and development phases, and ensures accurate and efficient added water and wood vinegar management.

### Hardware setup of the system

**Fig. 3:**
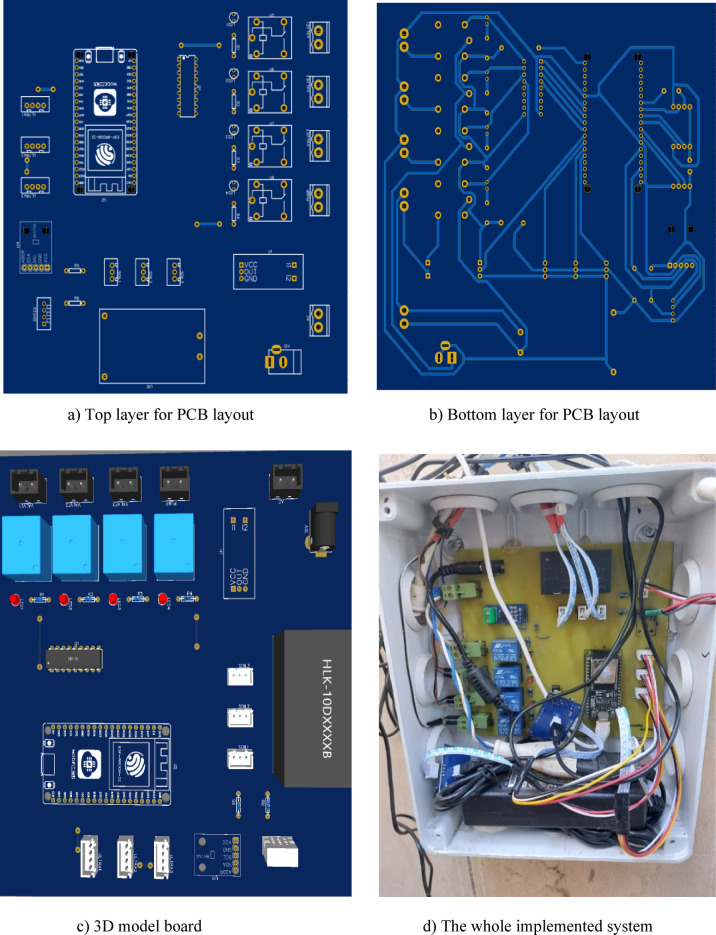
PCB layout (**a**,** b**), 3D model board (**c**) and the whole implemented system (**d**).

Design of printed circuit board (PCB) involves arranging all electronic components and their connections on a circuit board to create an efficient smart system. It is a crucial step in achieving the desired model, as it directly impacts the performance and reliability of the final system. The circuit diagram is translated into a physical representation on the board, specifying the locations of each component and how the copper wires route signals between them as shown in Fig. 3.

### Algorithm of smart irrigation system

**Fig. 4:**
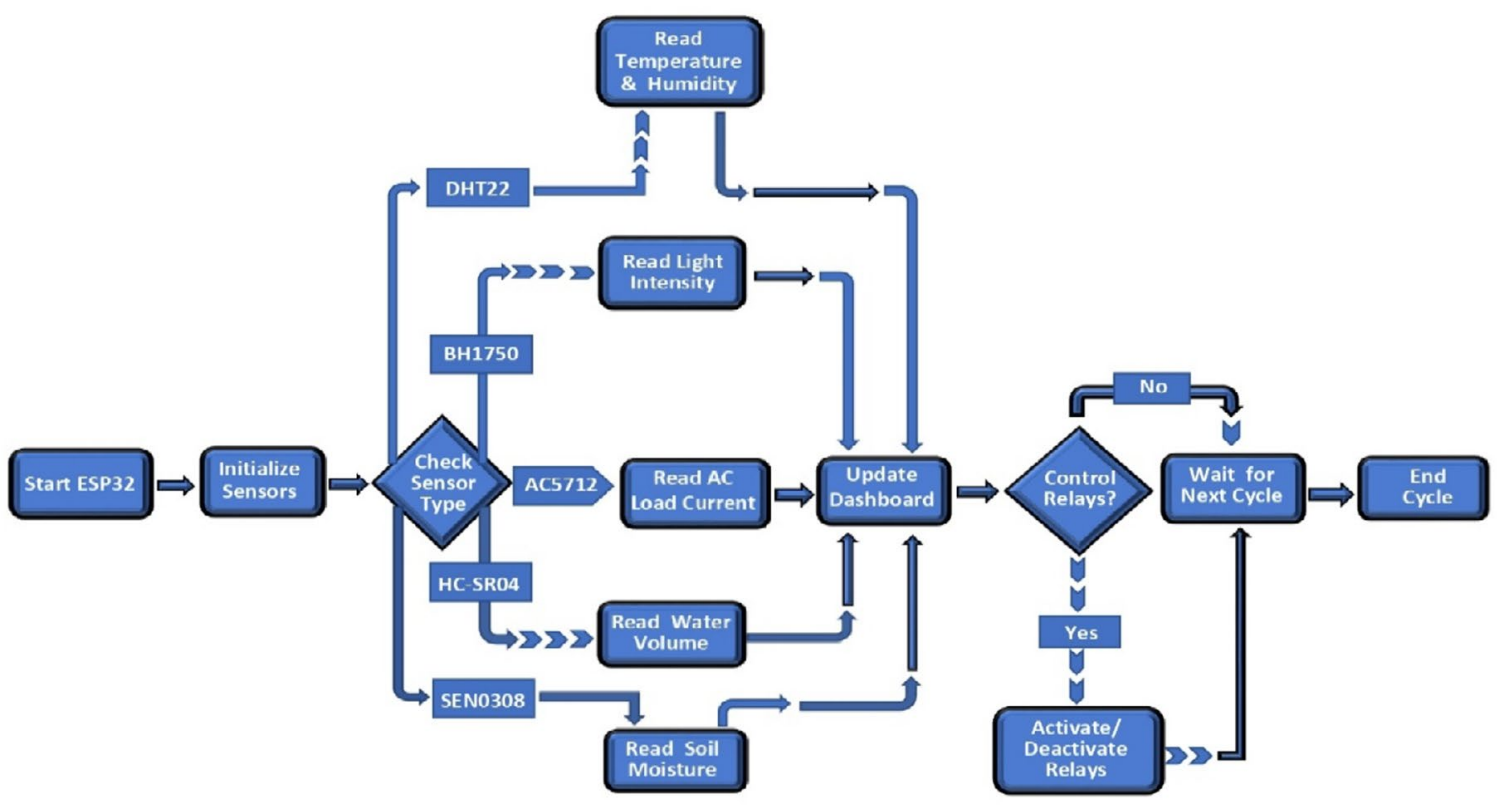
Algorithm of IoT-driven smart irrigation system.

The working steps for the algorithm of smart irrigation system can be showed in Fig. 4 as follows; Input: Login to Android application; All relevant soil and crop information from the drop-down menu and outputs: weather data and turning the irrigation pump on/off. Hence, the detailed steps of the smart irrigation system algorithm can be illustrated in the following points; (1) Continuously collect all sensors data through the microcontroller. (2) Save the sensors reading in the cloud server database. (3) Using the stored data, the model analyzes soil moisture sensor data to check whether drip irrigation is necessary or not. (4) Based on the recommendation of the sensor readings, the user will inform the ESP32-D0WD-V3 microcontroller board to turn on/off the irrigation pump. (5) The user can follow the recommendation and irrigate the plants.

### Programming code

The programming code describes the structure and function of a designed system to optimize operating conditions through real-time control and monitoring. Essential libraries for WIFI connectivity, TCP communication, and sensors management are involved, including the DHT library for temperature & humidity sensors. Table 3.

**Table 3.**
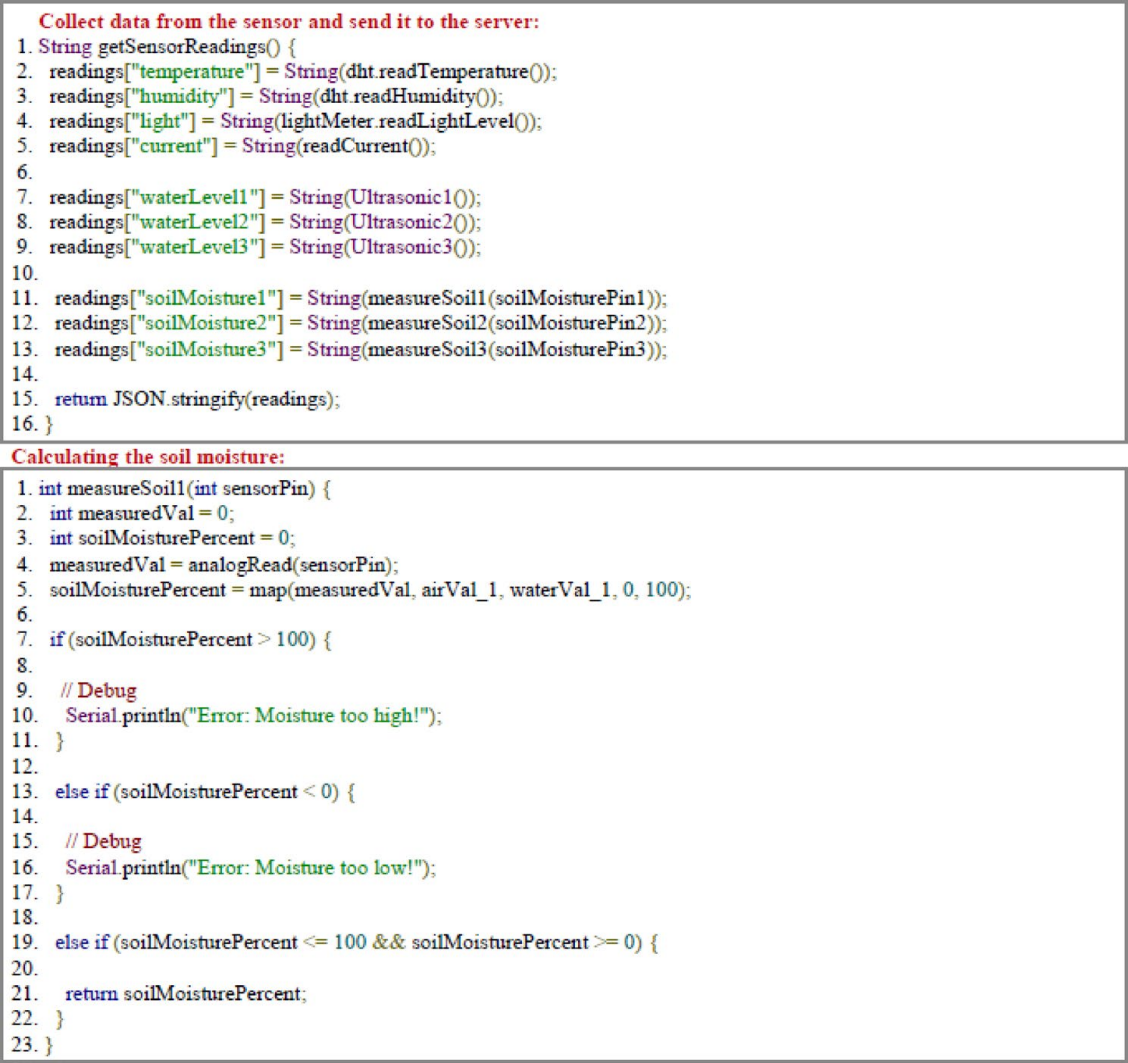

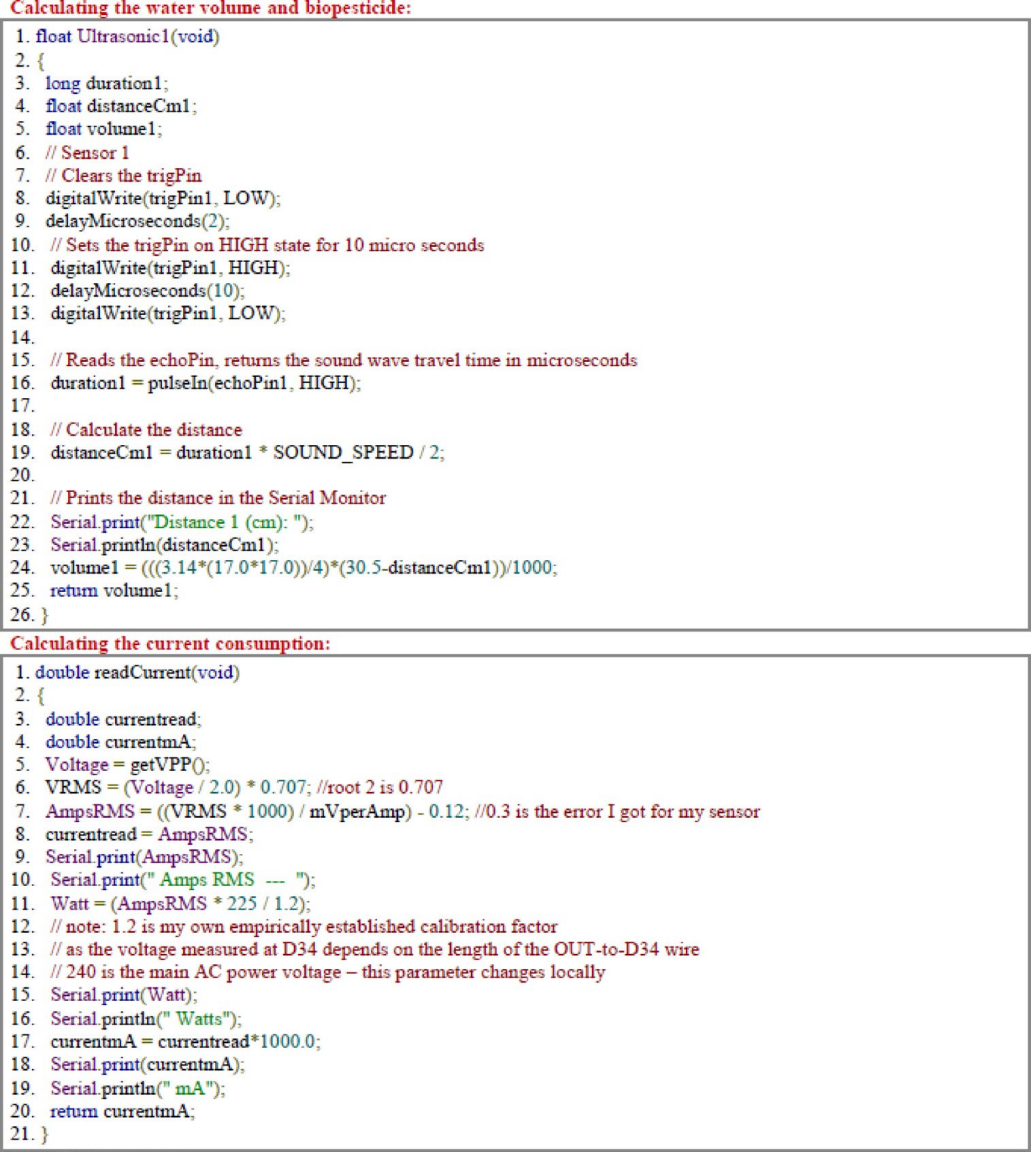
Input, process and output of the important functions.

### Data collection and measurements

A multiple sensor node is used to collect data in the specified area, depending on the experiment’s requirements. Data collection device consists of five sensors, soil moisture content (SEN0308), ultrasonic module (HC-SR04), temperature & humidity (DHT22), light intensity (BH1750), and current sensor ACS712. The output of these sensors is read by the microcontroller board ESP32-D0WD-V3, which is connected to the server through SPI/SDIO or I2C/UART interfaces. Programming code was written in Python language every hourly fetching the data from sensors, storing it the data in SQLite database and which synchronize with the server using a developed web service according to pre-defined algorithms. Four different concentrations of wood vinegar were used as treatments (T1-1:200, T2-1:300, T3-1:400, T4-1:500) at three soil moisture levels of 21, 26, and 32%. Water consumed (Wc), yield, water use efficiency (WUE), Leaf Area Index (LAI), Disease Severity Index (DSI) and power consumption were considered as evaluation criteria.

### Irrigation requirements

To estimate the total irrigation (total water volume) during plant growth, multiply the flow rate by the irrigation duration. Subtracting the volume of drainage collected under the pots calculates the amount of water consumed by the crop only. Using an ultrasonic sensor, the water volume is estimated, which first detects the water level and converts it to volume using modified software code in the smart system^16^.

### Yield and leaf area index

The plants were irrigated to physiological maturity based on the soil moisture level as measured by the sensors. Crop water use efficiency (WUE) was calculated using the following equations by^17^(Fernandez et al., 2020).

$$\:\mathbf{W}\mathbf{U}\mathbf{E}=\frac{\boldsymbol{Y}}{\boldsymbol{W}\boldsymbol{c}}$$

Where: Y is yield of crop (gm) and Wc is water consumed by the crop (l).

The leaf area index (LAI) of Lettuce is a dimensionless quantity representing the leaf area per unit of the ground area, is a key growth indicator, an important concept, and a valuable tool for improving production. LAI was calculated for Lettuce plant as follows^18^ (Mkhabela et al., 2019).

$$\:\mathbf{L}\mathbf{A}\mathbf{I}=\frac{\mathbf{L}\mathbf{A}}{\boldsymbol{G}\boldsymbol{A}}$$

Where; LA is the leaf area of all the Lettuce plants, cm^2^ and GN is the ground area cm^2^. The average area of leaf was calculated by measuring leaf length and width, then multiplying by a correction factor of 0.70, by randomly selecting three plants for each treatment.

### Disease severity index

Disease Severity Index, DSI is a measure used to determine the extent of disease symptoms on a Lettuce plant, typically based on the percentage of leaf area affected on the plant. A scale of 0 to 4 (for Fusarium Wilt) is used to evaluate the effectiveness of wood vinegar treatments and concentrations. It is calculated by multiplying each disease grade score by the number of plants with that grade, then dividing the result by the total possible score^19^.

$$\:\boldsymbol{D}\boldsymbol{S}\boldsymbol{I}=\frac{\left[{\boldsymbol{\Sigma\:}(\boldsymbol{f}}_{\boldsymbol{c}}\times\:{\boldsymbol{R}}_{\boldsymbol{c}})\right]}{\left[{\boldsymbol{P}}_{\boldsymbol{t}}\times\:{\boldsymbol{D}}_{\boldsymbol{i}}\right]}\times\:100$$

Where: f_c_ is class frequency, R_c_ is score of rating class, P_t_ is total number of plants and D_i_ is maximal disease index.

### Power consumption

In this study, it is determined the different operating modes of the communication sensors in the smart system. The first approach based on all electronic devices active during a specific time duration and inactive for a rest of the cycle. It should be noted that the sensors are mostly sleep mode in the time cycle. The consumed energy in this mode affects the amount of energy consumption of the sensors. All devices are supplied with the same voltage level 5VDC. After the current intensity is estimated by sensor ACS712, the power consumed (P) is calculated by the following equation:

$${\mathbf{P}}\,=\,{\mathbf{I\times{V}}}$$

Where: V is the voltage across each component (v) and I is the intensity of the current drawn through each electronic component (mA).

### Statistical analysis

The experimental data were analyzed using analysis of variance (ANOVA) based on Completely Randomized Design to assess the effects of treatment, moisture content, and their interaction on the studied parameters. The significance of differences among means was evaluated using the Least Significant Differences (LSD) test at a probability level of *P* < 0.01. Mean values are presented with their standard errors (mean ± SE). Statistical analyses were performed to assess both main effects and interaction effects, with significant differences identified by different letters assigned to group means (uppercase for main factors, lowercase for interactions).

## Results

This study presents a real-time system, designed for smart agriculture to enhance water use efficiency using advanced technology. Especially with regard to embedded systems, and optimized communication protocols. The proposed system is based on the use of the ESP32-D0WD-V3 microcontroller, which is connected with multiple sensors to measure soil moisture, water volume and current intensity as well as temperature and humidity and light intensity. The results indicate that both soil moisture levels and wood vinegar application had significant effects on all measured parameters, including actual irrigation (Ia), yield, water use efficiency (WUE), leaf area index (LAI), and disease severity index (DSI).

### Weather data

**Fig. 5:**
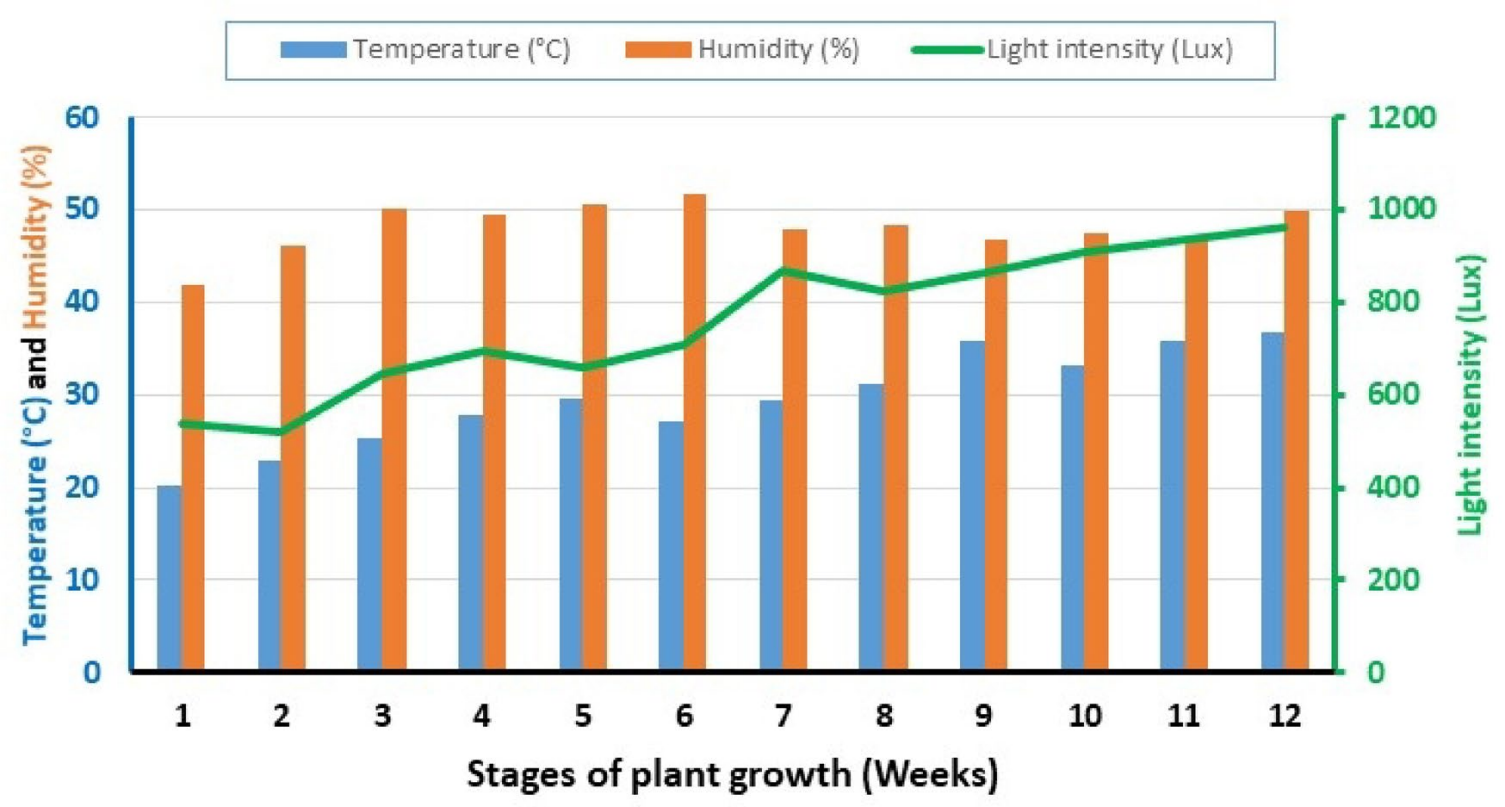
Ambient temperature, humidity and light intensity measured by (DHT22) and (BH1750) sensors during the growth stages of Lettuce.

The air temperature during the growth stages ranged from 20.33 to 36.83 ◦C, with an average of 29.64 ◦C as shown in Fig. 5, This range is suitable conditions for Lettuce plant. Temperature is an important factor affecting the growth and health of plant. While, the air humidity ranged from 41.97% to 51.60%, with an average of 48.10%, indicating the observed levels are sufficient to support optimal growth and productivity^20^. Light intensity values between 520.13 lx and 963.60 lx were also recorded during the growth stages with an average of 760.76 lux^21^. Lettuce plant is sensitive to temperature optimal growth occurs within specific ranges. High temperatures lead to bitterness and reduced yield.

### Irrigation water

**Fig. 6:**
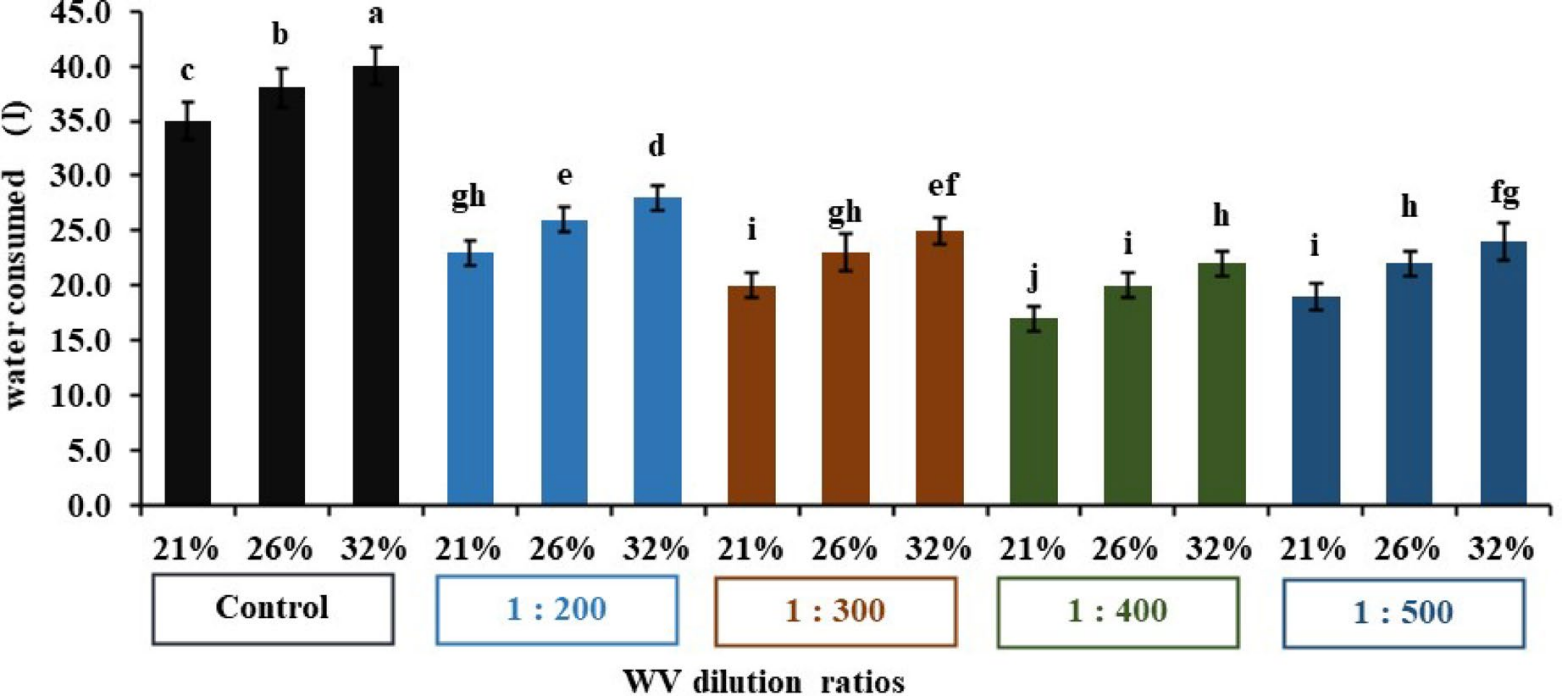
Impact of WV treatments on irrigation requirements under three moisture contents. The bars on the columns correspond to SE and different letters differ significantly by LSD (*p* < 0.01).

Significant interaction impacts between moisture content levels and wood vinegar dilution rates were observed for all studied traits (Fig. 6). Clearly, the impact of 400 treatment at 26% soil moisture content on water consumed surpassed that of 200 concentration and the control in saving water consumption. 400 treatment of WV significantly decreased the water consumed by 20 l, with respect to the control that had 38 l, representing a 47% reduction in water consumption based on actual irrigation. This system actively promotes water use efficiency and will prevent wasteful water usage^22^.

### Water use efficiency and LAI

**Fig. 7:**
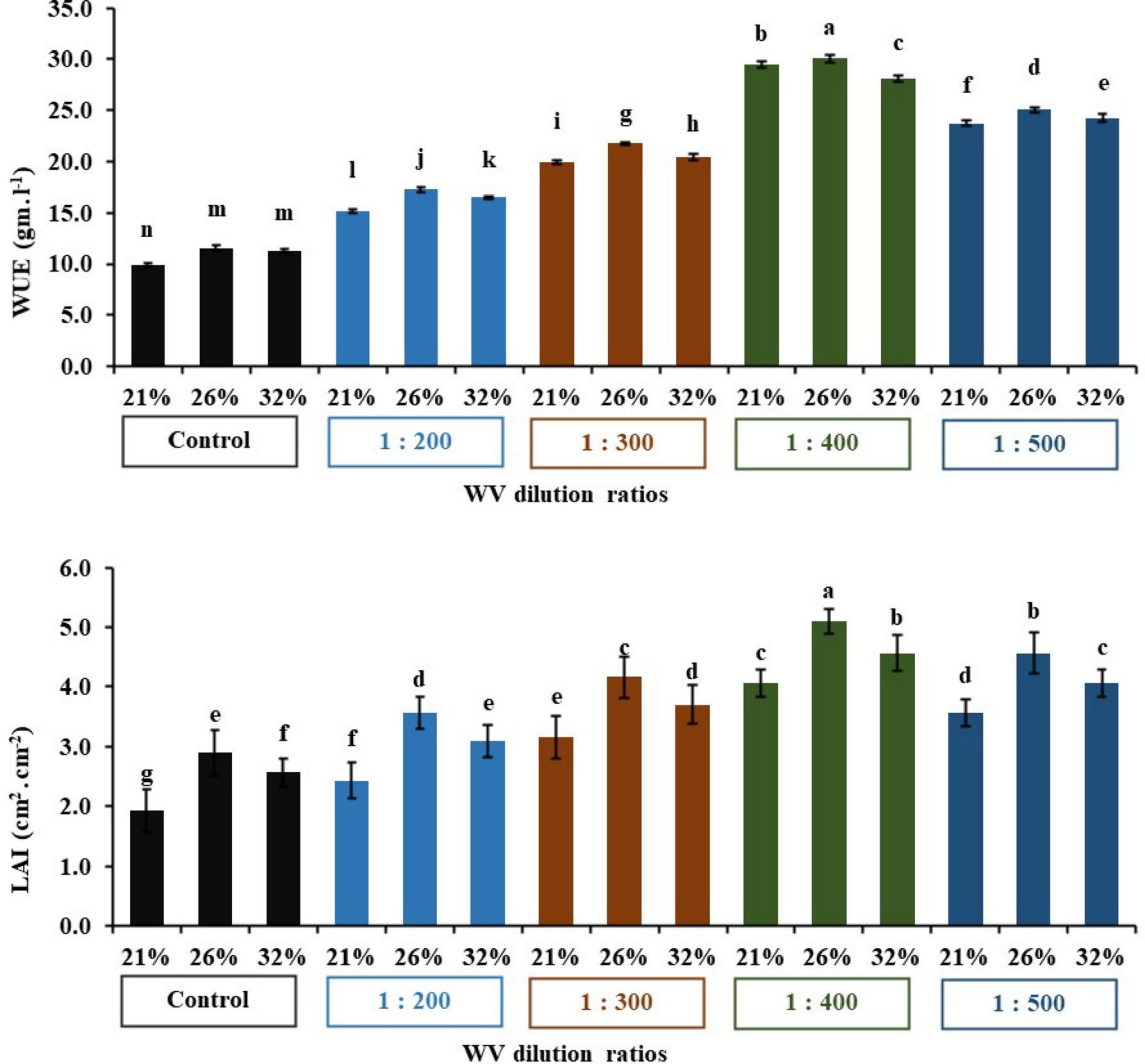
Impact of WV treatments on WUE and LAI under three moisture contents. The bars on the columns correspond to SE and different letters differ significantly by LSD (*p* < 0.01).

The obtained results evident that concentration of wood vinegar with 400 significantly (*p* < 0.01) promoted the Lettuce yield and leaf area index as presented in Fig. 7. This implies that 400 treatment produce superior water use efficiency and leaf area index by 30.1 gm.l^− 1^ and 5.1cm^2^.cm^− 2^, respectively under 26% soil moisture content compared to the control and other concentrations. Accordingly, it can be concluded that low concentrations of wood vinegar had a positive effect on Lettuce yield and leaf area index, which achieved a significant increase of 43% and 30%, respectively compared to other experimental treatments^23^.

### Disease severity index

**Fig. 8:**
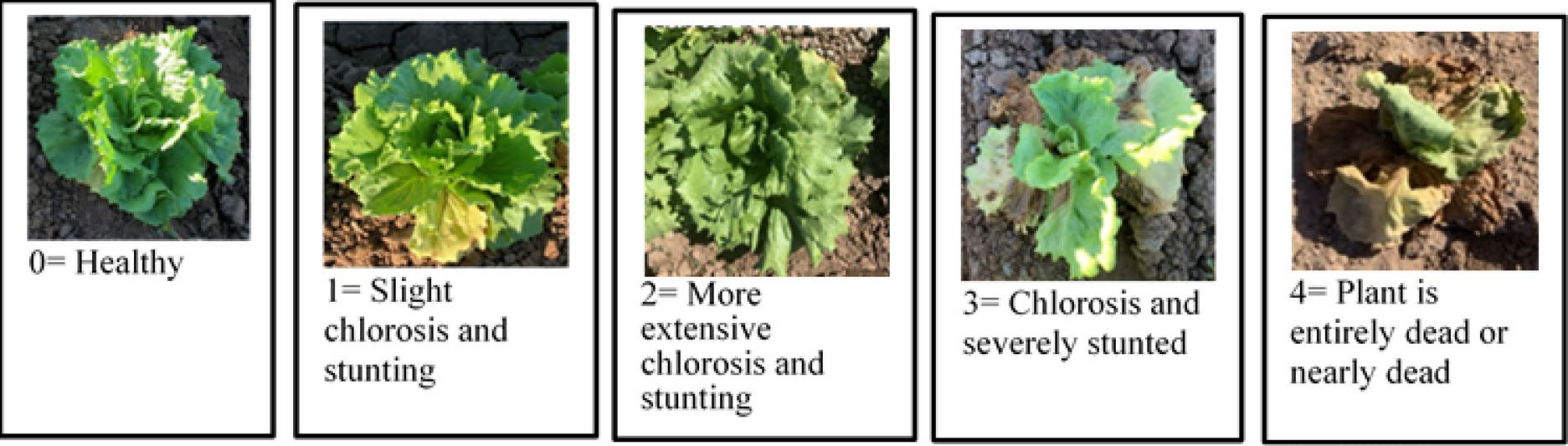
Disease severity rating.

Disease severity index (DSI) of fusarium wilt was evaluated on a 0 to 4 scale where: 0 = Healthy symptomless; 1 = wilting of one to three outer leaves; 2 = moderate stunting and wilting of < 25% of leaf area; 3 = head is severely stunted or absent and between 25% and 75% of leaf area is wilting; and 4 = > 75% of leaf area is chlorotic and nearly dead, or plant is entirely dead as shown in Fig. 8^4^.

**Fig. 9:**
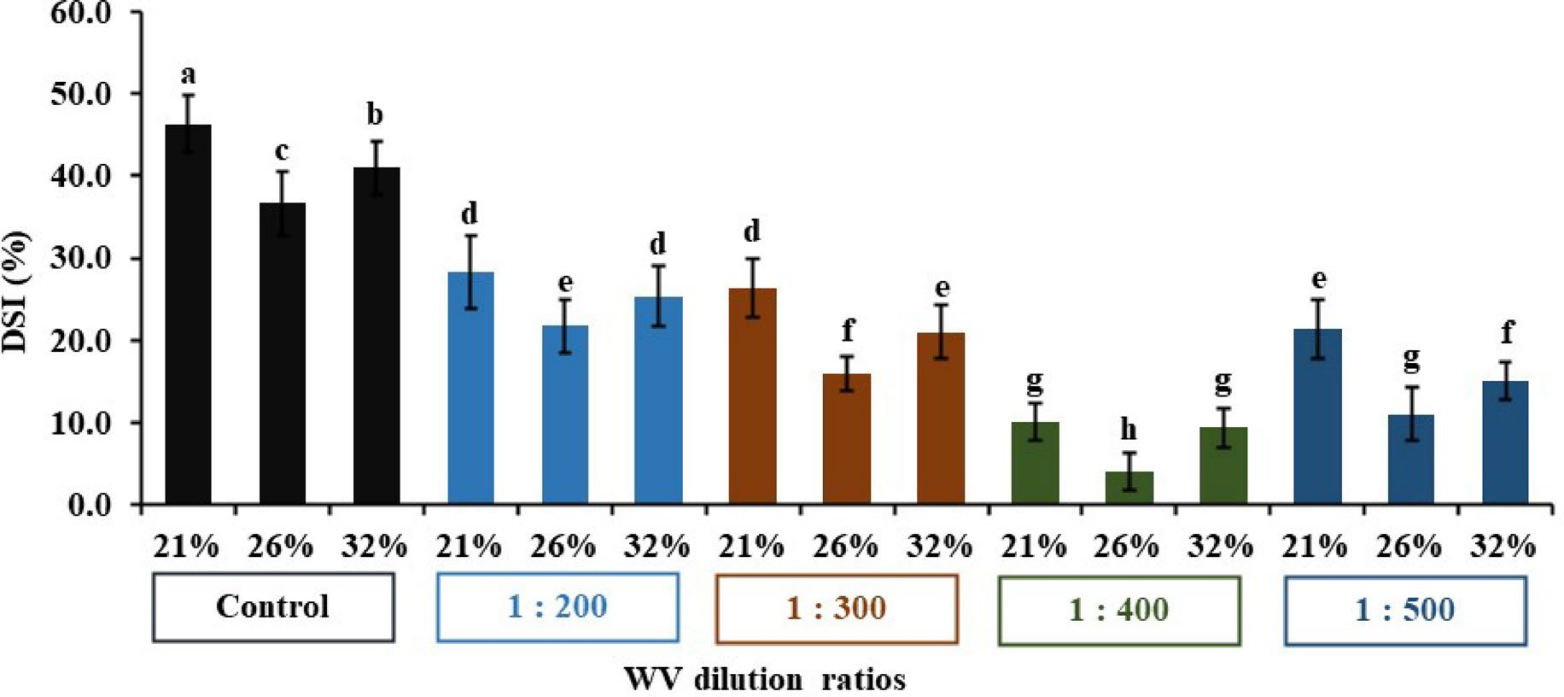
Impact of WV treatments on DSI under three moisture contents. The bars on the columns correspond to SE and different letters differ significantly by LSD (*p* < 0.01).

The analysis of variance indicated that the assessed treatment, moisture content, and their interaction had statistically significant effects on disease severity index (*P* < 0.01). The study included the effect of wood vinegar on Fusarium wilt for Lettuce plants. The highest DSI was assigned to the control, followed by the concentration of 200. While the lowest DSI was observed at the 400-treatment level with a value of 7.78%. The lowest DSI was observed at 26% soil moisture content as shown in Fig. 9.

The interaction between treatment and moisture content was also significant, highlighting that their combined influence plays a crucial role in evaluating DSI. Parameter varied significantly with the combination of treatments and moisture content. The lowest DSI occurred at 400 treatment and 26% moisture content, while the highest DSI was seen without treatment and 21% moisture content. The disease severity ratio was converted to marketability, where: disease severity ratio less than 25% marketable (0 and 1); ratio greater than 25% non-marketable (2, 3 and 4). Hence, WV concentrations of 400 and 500 were found to be marketable at rates ranging from 4% to 21%^24^. Wood vinegar has multiple roles, it acts as a natural fungicide that targets soil-borne and leaf-borne plant pathogens. In addition to its pesticidal properties, it contributes to soil health by regulating pH and stimulating beneficial microbial activity^15^.

### Power requirements

**Fig. 10:**
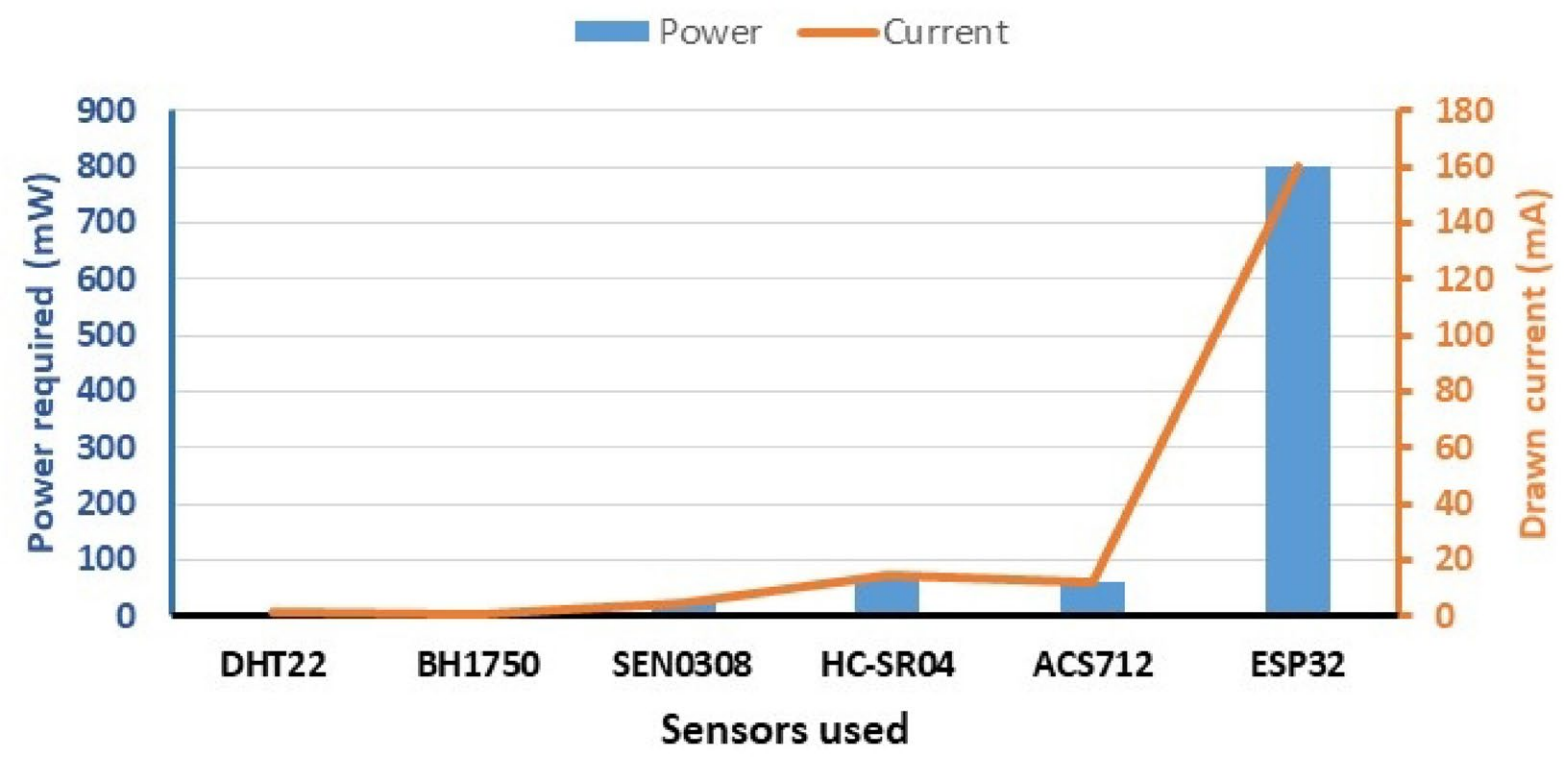
Consumed power and current intensity of smart system components.

Temperature & humidity (DHT22) sensor draws 1.5 mA of current during active mode and power of 7.5mW, light intensity sensor (BH1750) draws a low current of 0.12 mA and power of 5.0mW, Analog waterproof soil moisture sensor (SEN0308) draws power of 25mW and a current intensity of 5.00 mA in operating mode, ultrasonic sensor (HC-SR04) requires 15 mA of current to operate transmitter and receiver during the measurement cycle and power of 75mW, current sensor (ACS712) draws 12 mA of current during active mode and power of 60mW. While, microcontroller unit (ESP32) typically draws 160 mA of current and 800 mW of power in active mode and the water pump requires 200 W of power and 315 mA of current during operation as shown in Fig. 10. Hence, the total power consumed reached 200.97 W for all components of the smart system, including sensors and the control unit. Hence, the low power consumption of the smart system can be observed, making it a viable technology with minimal energy consumption^25^.

### Mobile application

**Fig. 11:**
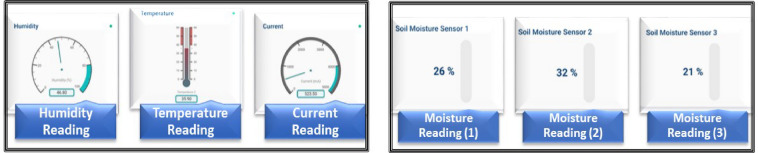
Real-time readings of temperature, humidity, current and soil moisture sensors on the mobile interface.

A mobile application is implemented to monitor and control the entire system by providing a user interface for real-time data visualization. This application allows users to manage and adjust smart system parameters and monitor system performance from their mobile devices. Environmental parameters can be monitored via the user interface over time reading of these parameters, as illustrated in Fig. 11 including temperature, humidity and soil moisture^23^.

**Fig. 12:**
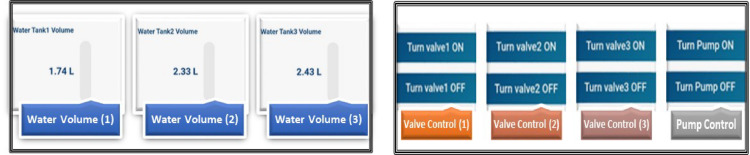
Real-time water volume readings and controlling dashboard of the irrigation valves and the pump for the smart system on the mobile phone interface.

In this menu, the system displays also the measurements of the water volume in real time using gauges as shown in Fig. 12, and the measurement history is displayed over time. In addition, there is also a menu that remotely controls the solenoid valves and water pump. There are 4 activation buttons in this menu can be set to “on”, 4 “off” buttons to turn off the valves and pump as illustrated in Fig. 12. Irrigation will be carried out according to the experimental treatments soil moisture^22^.

The results of this study will contribute to enhance the water use efficiency and provide agricultural management in the long term. The current study focused on exploring a new technology that ensures optimal use of resources, environmental sustainability, and effective management of irrigation which provides significant benefits to farmers.

## Discussion

IoT-driven smart system improves biopesticide application, taking into account implementing best practices of treatment to achieve the appropriate concentration. The study also focused on exploring a new technology that ensures optimal use of resources, environmental sustainability, and effective management of plant diseases which provides significant benefits to farmers. The results of this study will contribute to enhancing the capacity to sustainably manage its agricultural resources and provide agricultural management services in the long term. Wood vinegar has multiple roles, it acts as a natural fungicide that targets soil-borne and leaf-borne plant pathogens. In addition to its pesticidal properties, it contributes to soil health by regulating pH and stimulating beneficial microbial activity^15^.

It is also presented an advanced technology including embedded systems, HTTP, HTML, SSE and CSS. The system depends on the use of the ESP32 development board (WIFI - Bluetooth), connected to several sensors for measuring temperature and humidity, light intensity, soil moisture, water volume and current intensity. The results of this work contribute to to improve the capacity to manage water resources, increase of agricultural productivity as well as improving water and energy efficiency^6^. Irrigation water requirements expect to change with climate change. Therefore, it is necessary to monitor weather data regularly so that plants are not exposed to devastating effects if suitable environmental conditions are not available. Accordingly, the positive and effective role of the smart system can be concluded, especially in monitoring weather conditions during the plant growth stages^5^.

Lettuce plant is sensitive to temperature optimal growth occurs within specific ranges. High temperatures lead to bitterness and reduced yield. While lettuce tolerates low temperatures, extremely low temperatures slow vegetative growth. The humidity levels observed during the growth stages are quite suitable, with high humidity reduces transpiration rates, leading to leaf burn and reduced nutrient uptake. On the other hand, low humidity causes stress of the plant, especially when combined with high temperatures, this also led to tip burn and reduced growth. Light intensity is a crucial factor in lettuce growth, results in optimal fresh weight and productivity. While plant benefits from light, excessive intensity can cause leaf sunburn. Low light intensity can negatively impact lettuce growth and development. Plants grown under low light conditions may exhibit reduced leaf size and decreased chlorophyll content, leading to lower yields and reduced quality. It’s important to choose the optimal level^20^.

Accordingly, the positive and effective role of the smart system can be concluded, particularly in saving water consumption. Wood vinegar help reduce irrigation water usage by improving soil properties and enhancing plant health. Wood vinegar, at the appropriate concentration, plays a role in irrigation conservation by enhancing nutrient uptake, improving soil health, and reducing the need for chemical treatments. Generally, diluted wood vinegar solutions are more beneficial than concentrated ones^26^.

Wood vinegar reduce irrigation consumption by enhancing plants water use efficiency. This is achieved by improving nutrient uptake. The bioactive compounds (organic acids and phenols) enhance a plant’s ability on the absorption of essential nutrients. This leads to improve overall health of the lettuce plant and reduces the need for excessive irrigation process. It is also stimulated deeper root growth, allowing lettuce plant to access irrigation water from deeper soil layers. Wood vinegar helps lettuce plants better tolerate drought stress by activating their antioxidant defenses, mitigating the harmful effects of oxidative stress caused by lack of irrigation water^3^.

Smart system is determined irrigation requirements of lettuce plant during the stages of growth, based on measuring the moisture content using a soil moisture sensor (SEN0308), in addition to estimate the water volume using an ultrasonic sensor (HC-SR04), these accurate measurements contribute to determining the actual irrigation of crop in an optimal and timely manner, which reduces water losses^6,27^.

The obtained results manifested that both soil moisture levels and wood vinegar application had significant effects on all measured parameters for lettuce crop. WUE and LAI were highest at 75%-Fc and 90%-Fc, with the 75%-Fc level resulting in the highest WUE (19.02 gm.*l*^− 1^) and LAI (4.49 cm^2^.cm^− 2^). Both WUE and LAI decreased at 100%-Fc, with values of 14.17 gm.*l*^− 1^ and 3.73 cm^2^.cm^− 2^, consecutively.

The lowest Ia and DSI were observed at low moisture levels (22.58*l*) & (7.50%) at 60%-Fc level and (29.25*l*) & (6.17%) at 75%-Fc level, respectively. Actual irrigation (Ia) and disease severity index (DSI) increased as soil moisture increased, with the control (100%-Fc) showing a significantly higher values 42.08*l* and 29.08% in sequence. Figure 10. illustrates development stages of lettuce crop (seedling, rosette, cupping and heading).

Leaf area index is a reference tool for lettuce growth as leaves are the most important structure for photosynthesis process. It is aid in optimizing irrigation, fertilization, pest resistance, and other management practices. Wood vinegar promotes seed germination as it is a plant growth regulator and enhances seedling vigor^5^. It also supports vegetative growth and enhances resistance to both biotic and abiotic stresses. Wood vinegar significantly increase chlorophyll content and lettuce biomass particularly fresh weight at lower concentrations. Its use also leads to an increase in (LAI), by improving nutrient absorption and enhancing photosynthesis, which ultimately leads to increased crop productivity^12,28^.

Many researchers have confirmed the effectiveness of this technology in significantly increasing vegetative growth, plant height, biomass, dry weight and total productivity in the regulated deficit irrigation (RDI) system at 75% of the field capacity compared to full irrigation at 100%. In this study, four irrigation levels were evaluated (60, 75, 90, and 100) from the field capacity while using different concentrations of wood vinegar to study the effect of over- and under-irrigation conditions on pepper and lettuce yields and water use efficiency. Many researchers have concluded that over-irrigation failed to significantly increase yield compared to the appropriate irrigation level. In this work, the results showed that over-irrigation (90%- and 100%-FC) did not have a positive effect on all studied traits, but significantly reduced water use efficiency. Improving water use efficiency is urgent and necessary, especially in arid environments, to deal with limited water resources. Therefore, it is not recommended to increase irrigation quantities by more than 75%-FC at a 400-dilution rate of wood vinegar^10^. The optimum moisture content treatment resulted in a significant reduction in actual irrigation compared to other treatments, saving a 32% in water consumption. Hence, we conclude that this system significantly improves water use efficiency and prevents its waste.

The application of IoT-based irrigation improved productivity and saved water by 35% compared to traditional methods. These results clearly illustrate the potential of IoT-based systems to improve water use and increase crop yields, making them a viable option for sustainable agriculture. Applying moderate water stress (MWS) can stimulate the redistribution of photosynthetic products from slow-growing parts (leaves and stems) to more developed tissues, including (roots and fruits), thus enhancing water resource conservation without significantly compromising crop yields and thus improving water use efficiency. IoT-driven smart system improves biopesticide application, taking into account implementing best practices of treatment to achieve the appropriate concentration. The study also focused on exploring a new technology that ensures optimal use of resources, environmental sustainability, and effective management of plant diseases which provides significant benefits to farmers. The results of this study will contribute to enhancing the capacity to sustainably manage its agricultural resources and provide agricultural management services in the long term. Wood vinegar has multiple roles, it acts as a natural fungicide that targets soil-borne and leaf-borne plant pathogens. In addition to its pesticidal properties, it contributes to soil health by regulating pH and stimulating beneficial microbial activity^15^.

Regarding hardware components, the current system is characterized by its low energy consumption, which is especially important for microcontroller unit, sensors, and networked IoT devices that rely on limited energy sources as a result of using low voltage and current drawn in all electronic devices and units, which reduces energy costs and promotes environmental sustainability. It was taken into account to write efficient programming codes, which reduces processing time and memory usage in terms of software design. Energy-aware scheduling was used, through which tasks can be prioritized based on their energy requirements and scheduled. IoT technology in this system can utilize optimized communication protocols like HTTP, HTML, CSS and JavaScript, which are designed to be energy-efficient. These protocols minimize the amount of data transmitted and the energy consumed during communication process^29^.

## Conclusion and future work

This study presents a smart real-time system, specially designed for improving irrigation management, using advanced technologies like IoT, embedded systems and sophisticated communication protocols. The proposed system is based on the use of the ESP32-D0WDR2-V3 microcontroller connected to several sensors, including soil moisture content, ultrasonic module, temperature & humidity, light intensity and current sensor. The obtained results contribute to improve water use efficiency and increasing agricultural yield, which enhances the ability to sustainably water resources management and provide agricultural services in the long term. The results show that, by virtue of smart irrigation management, the system is able to maintain optimum soil moisture the threshold of 26%, while a 400-dilution rate of wood vinegar. The smart system also reduces water consumption by 47% and achieving a 43% increase in yield as well the lowest level of disease severity index with a value of 7.78%, thus contributing to a more sustainable management of water resources with improved practices of the precision farming. The proposed system enables continuous monitoring and faster, more accurate decision-making, which is critical in light of climate change and increasing weather variability. The integration of climate data and soil parameters also enhances better prediction of crop needs, ensuring optimal management of agricultural resources^30–33^.

In addition to the aforementioned prospects, the integration of renewable energy is a key avenue for enhancing energy sustainability. Using photovoltaic system will enable sensors and IoT devices to operate autonomously, reducing reliance on conventional energy sources and improving the system’s viability in resource-limited environments. Advances in artificial intelligence, Internet of Things (IoT) sensors, autonomous tractors, and drones for field monitoring and data analytics will also shape the future of precision agriculture in the face of future environmental and technological challenges. Thus, our future work plans to enhance secure transmission protocols, implement encryption mechanisms to ensure information protection and data security, reduce overall costs, and educate farmers. This will lead to improved efficiency, increased yields, and environmental sustainability.

## Data Availability

The data that support the findings of this study are available from the corresponding author upon reasonable request.
